# Supplementary material for: Aggregation of Mouse Serum Amyloid A Protein Was Promoted by Amyloid-Enhancing Factors with the More Genetically Homologous Serum Amyloid A
Source: Int J Mol Sci. 2021 Jan 21;22(3):1036. doi: 10.3390/ijms22031036 (PMC7864520; doi:10.3390/ijms22031036)
Supplement: Supplementary file 1 [file ijms-22-01036-s001.pdf]

International Journal of *Molecular Sciences*

MDPI

Article

Aggregation of mouse serum amyloid A protein was promoted by amyloid-enhancing factors with the more genetically homologous serum amyloid A

Xuguang Lin [1], Kenichi Watanabe [2], Masahiro Kuragano [1] and Kiyotaka Tokuraku [1,*]

1 Graduate School of Engineering, Muroran Institute of Technology, Muroran, Japan
2 Department of Veterinary Medicine, Research Center of Global Agromedicine, Obihiro University of Agriculture and Veterinary Medicine, Obihiro, Japan
* Correspondence: Corresponding: E-mail: tokuraku@mmm.muroran-it.ac.jp Tel: +81-0143-46-5721.

Supplemental Figures

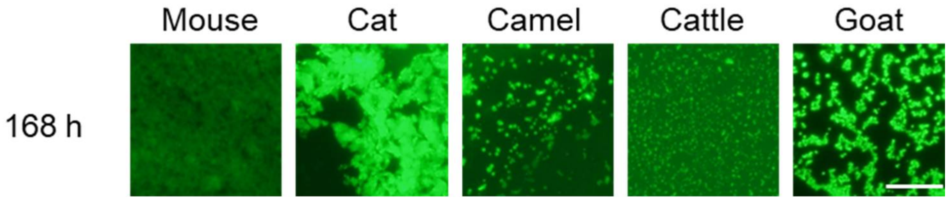

Figure S1. Imaging of mouse serum amyloid A aggregates by Thioflavin T in the presence of 100% amyloid enhancing factor after 168 h of incubation. Similar to the quantum dots imaging results (Figure 3A, 100%), the mouse sample showed a mesh-like form and otherwise dot-like forms. In addition, a large amount of aggregates was observed in the cat sample. Scale bar in fluorescent micrograph indicates 100 μm.
